# Prácticas de educación permanente en atención primaria a la salud para el abordaje de personas usuarias con tuberculosis

**DOI:** 10.18294/sc.2023.4542

**Published:** 2023-11-23

**Authors:** Letícia Vieira Lourenço, Karla Santa Cruz Coelho, Emerson Elias Merhy

**Affiliations:** 1 Magíster en Atención Primaria de la Salud. Coordinadora pedagógica, Programa de Residencia en Enfermería Familiar y Comunidad, Secretaría Municipal de Salud, Rio de Janeiro, Brasil. leticiavlourenco@gmail.com Universidade Federal do Rio de Janeiro rograma de Residencia en Enfermería Familiar y Comunidad Secretaría Municipal de Salud Rio de Janeiro Brasil leticiavlourenco@gmail.com; 2 Doctora en Salud Colectiva. Profesora asociada, Universidade Federal do Rio de Janeiro, Campus Macaé, Río de Janeiro, Brasil. karlasantacruzcoelho@gmail.com Universidade Federal do Rio de Janeiro Universidade Federal do Rio de Janeiro Campus Macaé Río de Janeiro Brazil karlasantacruzcoelho@gmail.com; 3 Doctor en Salud Colectiva. Profesor Titular, Universidade Federal do Rio de Janeiro, Campus Macaé, Río de Janeiro, Brasil. emerhy@gmail.com Universidade Federal do Rio de Janeiro Universidade Federal do Rio de Janeiro Campus Macaé Río de Janeiro Brazil emerhy@gmail.com

**Keywords:** Fecundación in Vitro, Edición Genética, Ectogénesis, Técnicas Reproductivas, Bioética, Fertilization in Vitro, Gene Editing, Ectogenesis, Reproductive Techniques, Bioethics

## Abstract

El creciente campo de la reproducción humana asistida ha alcanzado hitos inimaginables. Su continuo desarrollo y las innovaciones que genera, en ocasiones, plantean dilemas tanto éticos como jurídicos. El presente ensayo trata de exponer los cambios progresivos que se están viviendo en el ámbito del origen de la vida debido al desarrollo de nuevas opciones y estrategias en reproducción humana asistida. En primer lugar, se realiza una reflexión interdisciplinar desde la ciencia, la ética y el derecho, sobre la naturaleza humana y los cambios a los que la sociedad se enfrenta, en particular, desde la perspectiva española. En segundo lugar, recoge una breve aproximación en torno a las técnicas biomédicas presentes o futuras en el campo de la reproducción humana. Concluye sobre la necesidad de reflexionar ante el vertiginoso avance de la ciencia en materia de reproducción humana asistida.

## Abstract (en)

The growing field of assisted human reproduction has achieved unimaginable milestones. Its continuous development and the innovations it generates at times pose both ethical and legal dilemmas. This essay aims to elucidate the progressive changes occurring in the realm of the origin of life due to the development of new options and strategies in assisted human reproduction. First, it constructs an interdisciplinary reflection on human nature and the changes society faces from the perspectives of science, ethics, and law, particularly from the perspective of Spain. Second, it provides a brief overview of current or future biomedical techniques in the field of human reproduction. It concludes with a discussion of the need to reflect on the rapid advancement of science in assisted human reproduction.

## INTRODUCCIÓN

La tuberculosis es una enfermedad transmisible, que representa la principal causa de muerte por un único agente infeccioso conocido, solo superada por el VIH-sida^1^. Sin embargo, esta posición cambió en abril de 2020, cuando el covid-19 la superó en términos del número de muertes diarias^2^; y, actualmente, representa la segunda mayor causa de muertes por enfermedades infecciosas después del covid-19^1^.

Antes de la pandemia de covid-19, muchos países mostraban constantes progresos en la lucha contra la tuberculosis. Los datos epidemiológicos indican una reducción del 9% en la incidencia entre 2015 y 2019 y una disminución del 14% en las muertes por la enfermedad en el mismo período. Y a partir de 2020 es posible observar un aumento de la tasa de pobreza extrema global por primera vez en más de 20 años^3^.

El impacto negativo en los indicadores de tuberculosis puede explicarse, en parte, por las altas demandas de salud generadas por la pandemia ya que, en muchos países, los recursos humanos y financieros, entre otros, fueron reasignados para dar respuesta al covid-19, lo que llevó a interrupciones en los servicios de salud. Esto se debe a que el acceso equitativo al diagnóstico, prevención, tratamiento y cuidados de calidad y oportunos se vio comprometido^3^, lo que llevó a que, en el año 2021, por primera vez en más de una década, las muertes por la enfermedad aumentaron, de acuerdo con el informe global de la Organización Mundial de la Salud (OMS)^4^.

En Brasil, la Red de Atención a la Salud (RAS) sigue las pautas del Sistema Único de Salud (SUS)^5^, por lo que la atención primaria de la salud constituye el primer nivel de atención a la salud. Entre sus directrices, se destacan acciones para la organización del cuidado como, por ejemplo: “practicar el acogimiento en todas las relaciones de cuidado, tanto en los encuentros entre trabajadores de salud y las personas usuarias, como al evaluar el riesgo y vulnerabilidad de las familias del territorio”; “desarrollar relaciones de vínculo y responsabilidad entre el equipo y la población de su territorio de actuación”; y llevar a cabo acciones de cuidado construidas junto con las personas, de acuerdo con sus necesidades y potencialidades^6^.

En los procesos de trabajo desarrollados en atención primaria existen muchos desafíos a ser superados. Esto se debe a que, para cumplir con la directriz de producir cuidado centrado en la persona, es necesario calificar diversos aspectos que van desde comprender las líneas de cuidado como organizadoras del trabajo, fomentar la vinculación de los equipos de salud con la población de la región en la que se sitúan y actúan, hasta la oferta de procedimientos alineados a las especificidades de la persona usuaria, sus necesidades y su singularidad^7^.

En el abordaje de personas con tuberculosis en el marco de la atención primaria, la “interrupción del tratamiento” constituye uno de los obstáculos para el control de la enfermedad. En este artículo, con el fin de alinear la discusión con las directrices del lenguaje centrado en la persona y de evitar el uso de palabras estigmatizantes, el término “abandono” será sustituido por la expresión “interrupción del tratamiento”, al reconocer que la persona usuaria-ciudadana no es la principal responsable del resultado desfavorable del tratamiento. Tal visión demanda implementar una metodología de prácticas del cuidado en salud que afecte a todos las personas involucradas^8^. La apuesta es que los equipos elaboren soluciones adecuadas, de forma compartida con los diversos actores. Se espera que cuanto mayor sea su proximidad y su conocimiento de los problemas enfrentados por los habitantes del territorio, sus necesidades, demandas y potencialidades, mayor sea la posibilidad de ampliar el campo del cuidado.

Entre 2016 y 2020, en Brasil se observó un aumento en la proporción de personas que interrumpieron el tratamiento, del 20,2% (n=223) al 22,9% (n=189^8^. Este desenlace presenta implicaciones como el mantenimiento de la cadena de transmisión, pues aquellas personas que no adhieren satisfactoriamente a la terapia medicamentosa continúan siendo fuente de contagio. Esta cuestión tiene implicancias también en la resistencia farmacológica, posterga la cura, encarece el tratamiento, aumenta la duración y la gravedad de la enfermedad, eleva las tasas de mortalidad, además de tener un importante impacto económico, tanto para las personas usuarias, como para el sistema de salud^9^.

De modo general, en la bibliografía se describen factores asociados a la interrupción del tratamiento relacionados con el usuario y con los servicios de salud. Sin embargo, las normativas propuestas por las políticas de salud, a veces se muestran poco flexibles, y, por consiguiente, tienden a invisibilizar las dificultades experimentadas por las personas, en su mayoría, habitantes de favelas, que residen en espacios superpoblados y sin oportunidad de empleo formal^10^. Como consecuencia, las dificultades enfrentadas por los usuarios para asistir a las consultas, mantener el tratamiento regular o realizar los exámenes, no siempre son identificadas y comprendidas por el equipo de salud.

Las dificultades para identificar y trabajar los factores subjetivos que llevan a la interrupción del tratamiento pueden ser entendidas a la luz de las relaciones establecidas en los encuentros entre la persona usuaria y el personal de salud. Esto se debe a que, desde la perspectiva de la organización de los servicios de salud, percibimos la profunda crisis del paradigma que estructuró el modo actual de trabajar del médico, marcado por el predominio de las modalidades de intervención centradas en las tecnologías duras, a partir de un conocimiento estructurado reducido a la producción de procedimientos^11^.

La expresión “tecnologías” ha sido utilizada en el campo de la salud para designar la sistematización de los diversos modos de producir salud y, según el tipo de tecnología que presida el encuentro, mayor o menor será su potencial cuidador, la porosidad para el encuentro, y el intercambio y la construcción de sentidos comunes^12^. Este modo de operar el trabajo centrado en las tecnologías duras genera una relación usuario-trabajador costosa y ocasionalmente resolutiva, y marcada por un proceso de alienación y de desresponsabilización^11^.

En la discusión sobre el abordaje de la persona con tuberculosis es importante tener en cuenta que el objeto no es la cura o la promoción y protección de la salud, sino la producción del cuidado^13^. Pues incluso ante los casos de usuarios en los que no se obtenga la cura y, por consiguiente, el alta con el tratamiento completo, siempre hay posibilidades de gestionar acciones de cuidado. De este modo, el trabajo se torna desafiante y de intensa producción, y requiere de nuevas formulaciones centradas en el colectivo que allí habita. Pueden pensarse, por ejemplo, acciones de reducción de daños, sea por la manifestación de algún síntoma clínico, o por el uso de alcohol y otras drogas, o por la identificación y fortalecimiento de la red de apoyo de la persona usuaria en cuestión.

Sin embargo, es necesario comprender que la creatividad para trazar abordajes terapéuticos que promuevan la adhesión de la persona usuaria al tratamiento de tuberculosis puede no siempre ser factible. Esto se debe a que, las prácticas de cuidado de la Estrategia de Salud Familiar (ESF) tienen un carácter fuertemente burocrático, que disciplina al trabajador y limita su capacidad creativa como, por ejemplo: los protocolos estandarizados, la lógica de producción por procedimientos y la agenda orientada a los programas nacionales^14^^,^^15^.

Desde esta perspectiva, resulta necesario reformular las concepciones de formación de las y los trabajadores, para que se garantice no solo la innovación y los cambios institucionales, sino también la participación de las y los profesionales en la identificación y resolución de problemas. Para ello, el desarrollo de este artículo se sustenta tanto en el campo de la educación permanente en salud, que incentiva nuevas posturas sobre el cuidado de la salud^16^, como en uno de sus fundamentos centrales: la micropolítica del trabajo vivo, que reconoce el mundo del trabajo como un espacio de creación de nuevas subjetividades esenciales para el cambio institucional^16^^,^^17^.

El escenario de los servicios de salud configura espacios estratégicos para desencadenar nuevos procesos de producción de salud, nuevos desafíos para prácticas y conceptos dominantes y nuevas relaciones de poder. El campo de la educación permanente en salud constituye un camino para la construcción de este movimiento^18^, dado que implica la producción de conocimientos en el día a día de las instituciones de salud, a partir de la realidad vivida por los actores involucrados, donde los problemas del trabajo cotidiano y las experiencias de esos actores son la base de interrogación y de cambio, y la interrupción del tratamiento en tuberculosis un desenlace que dificulta el efectivo control de la enfermedad en Brasil y en el mundo^19^.

El análisis de los procesos micropolíticos cotidianos de los profesionales de la salud es esencial para identificar las oportunidades de transformación a nivel de la atención primaria de salud^20^. Esta transformación debe alejarse del modelo médico-hegemónico y orientarse hacia una ética comprometida con la vida, adoptando una postura acogedora, estableciendo vínculos, buscando soluciones y fomentando la autonomía de los usuarios. Estas características son fundamentales para mejorar las acciones de cuidado dirigidas a usuarios que han interrumpido el tratamiento de la tuberculosis.

Este artículo es el resultado de una investigación realizada en colaboración entre la Universidad Federal de Río de Janeiro, Campus Macaé, y el Programa de Control de la Tuberculosis de la Secretaría de Salud del Estado de Río de Janeiro (SES-RJ). El estudio buscó comprender los aspectos centrales que promueven el desarrollo de acciones centradas en las personas usuarias-ciudadanas, con el objetivo de que los tratamientos se completen sin interrupciones, lo que llevaría a una disminución de la transmisibilidad y la mortalidad por tuberculosis. Para ello, se reafirma la estrecha relación entre la tuberculosis, las condiciones de vida y el acceso a los servicios de salud, lo que hace que el proceso de salud-enfermedad-cuidado sea un fenómeno multicausal.

El objetivo de esta investigación es analizar qué prácticas de educación permanente en salud están siendo desarrolladas por los equipos de salud de la familia en las unidades de salud de Maré, ubicadas en el municipio de Rio de Janeiro, en el estado de Río de Janeiro, Brasil, en casos de interrupción del tratamiento de tuberculosis. A partir de las situaciones vividas en el trabajo vivo en acto, se busca dar visibilidad a la maraña de líneas y planos que configuran los recorridos terapéuticos alternativos a los cuidados estructurados en la cotidianidad, con el fin de garantizar la adhesión y, consecuentemente, la cura.

Creemos que la construcción colectiva del saber, a partir de la reflexión sobre los procesos de trabajo cotidianos, abre camino para mejorar la relación de cuidado establecida entre personas (profesional y usuaria). La educación permanente en salud, al promover el aprendizaje en el trabajo, actúa como una potencia transformadora frente a los diversos modos “conservadores” con los que se han constituido las prácticas de cuidado a lo largo de los años^21^, por lo que permite trazar nuevas acciones hacia un cuidado universal e integral para las personas usuarias que necesitan de los servicios de salud. 

## MÉTODOS

Se trata de una investigación exploratoria de naturaleza cualitativa que adoptó el flujograma analizador y la cartografía como orientación metodológica. La comunidad de Maré, ubicada en el municipio de Río de Janeiro, fue seleccionada como campo de investigación y el estudio investigó las prácticas de atención de las y los profesionales de la salud en el trabajo diario de las unidades de atención primaria de salud, en relación con el manejo de pacientes que abandonaron el tratamiento de tuberculosis.

El escenario elegido cuenta con 135.989 habitantes en toda la zona, siendo la región de la ciudad con la mayor cantidad de residentes en favelas. Es más grande que el 96% de los municipios brasileños, lo que lo convierte en una ciudad de tamaño mediano, y representa más del 9% de la población residente en favelas en el municipio de Río de Janeiro^22^.

El territorio cuenta con siete unidades de atención primaria que suman 36 equipos de salud, cada uno compuesto por un médico o una médica (se destaca la dificultad para cubrir esta posición, incluso con el incentivo financiero proporcionado por la Secretaría Municipal de Salud para retener a estos profesionales en el territorio). Además, cuenta con un enfermero o una enfermera, un personal técnico en enfermería y de cuatro a cinco agentes comunitarios de salud. Cada equipo es responsable de un número específico de calles y, teniendo en cuenta las personas registradas, cubre entre 3.500 a 4.000 personas.

Esta investigación es el resultado de una tesis de maestría, cuya autora e investigadora integraba uno de los equipos de salud en Parque União, ubicado en el conjunto de favelas de Maré. Ser enfermera de la comunidad en ese momento permitió la cercanía a través de la red de relaciones con otros profesionales de la salud, lo que facilitó el acceso a los territorios y el conocimiento en profundidad de la rutina de los servicios de salud. Por lo tanto, los participantes estaban al tanto de la trayectoria profesional de la investigadora, así como de las razones que llevaron al desarrollo de la investigación.

La investigación contó con la participación de doce profesionales de la salud de diversas categorías, de las siete unidades de atención primaria de salud que atienden a la comunidad. En Maré, todas las unidades tienen el mismo horario de funcionamiento, de 7:00 a 18:00 horas, y enfrentan dificultades debido a conflictos armados y tráfico de drogas, así como las escasas posibilidades de retener a médicos y médicas debido a la distancia de las áreas más céntricas y seguras de la ciudad y a la violencia que afecta la región.

De las siete unidades, dos se denominan Centros Municipales de Salud ya que, además de los equipos de atención primaria, cuentan con consultorios de especialidades, como ginecología e infectología. Aunque dan cobertura a territorios diferentes, estos se encuentran dentro del conjunto de favelas de Maré, que presenta características de viviendas densamente pobladas, servicios de saneamiento básico precarios y una población de edad similar.

Al entrevistar a profesionales de los equipos de salud en el servicio, la investigación buscó identificar las formas o líneas invisibles a través de las cuales brindan atención de salud a las personas usuarias que han interrumpido el tratamiento de tuberculosis. La elección del método de cartografía busca acceder al plano relacional de la micropolítica del trabajo en el ámbito de la salud, de manera que la propia persona que investiga afecta y se ve afectada por el campo de la investigación, en una interferencia recíproca, ya que implica la intensa producción de encuentros y afectos^23^.

La cartografía se produce a partir de la identificación de problemas detectados o potenciales, mapeados en cada situación presentada, que en esta investigación se realizó a partir de preguntas elaboradas y aplicadas mediante un cuestionario con siete preguntas abiertas. El objetivo fue identificar los conflictos y las tensiones que operaban en ciertas situaciones, las formas en que los sujetos se encontraban y se validaban, así como sus implicaciones en el acto, tanto consigo mismos como con los demás^18^.

Entre los recursos utilizados para recopilar los datos por parte de la investigadora enfermera se encuentra el uso de un diario de campo para registrar sus impresiones durante las visitas. También se emplearon grabaciones de audio para registrar los discursos.

Como criterio de selección de las y los participantes se utilizó el muestreo por conveniencia. La inclusión en la investigación requería que el o la participante fuera profesional de un equipo de atención primaria de salud y que estuviera de acuerdo con los términos del consentimiento informado. Se excluyeron profesionales que estuvieran en período de vacaciones en el momento de la recopilación de datos y que no aceptaran firmar el término de consentimiento informado. Un factor limitante fue la escasez de personal médico y la alta rotación de esta categoría en las unidades de salud. Esto se sumó a la carga de trabajo, especialmente durante el período de la pandemia en el que se recopilaron los datos por lo que, al momento de ser invitadas, no pudieran participar en la investigación.

Se contactó a las y los participantes por teléfono y se les explicaron las motivaciones e intereses de la investigación. Las entrevistas se llevaron a cabo entre agosto y noviembre de 2021. La investigación contó con la participación de profesionales de la salud de diversas categorías de las siete unidades de atención primaria de salud que atienden a la comunidad de la Maré. La muestra quedó conformada por 12 personas que trabajan en los equipos de estas unidades (cinco enfermeras, un enfermero, cuatro agentes comunitarias, dos técnicas de enfermería y un farmacéutico), quienes pudieron y estuvieron dispuestos a colaborar en la investigación y firmaron los términos del consentimiento informado. Los datos se recopilaron después de que el proyecto fuera sometido al Comité de Ética, siguiendo el protocolo CAAE: 40957020.90000.5699, cumpliendo así con la Resolución No. 510/2016, que regula la investigación con seres humanos^24^.

Una vez que los participantes aceptaron los términos de consentimiento libre e informado de forma voluntaria, se realizaron las entrevistas utilizando un guion de siete preguntas abiertas. 

Posteriormente, se transcribieron y se agruparon en bloques de discurso, considerando los relatos que estuvieran directamente relacionados con el objeto de estudio, en particular aquellos más recurrentes, lo que permitió construir una lectura comprensiva y crítica, respaldada por los registros del diario de campo de la investigadora. Para organizar e identificar los relatos, conservando la confidencialidad de las personas entrevistadas, se utilizaron nombres relacionados con la comunidad, como: caracol, agua, mar, playa, pez, ostra, ola, sal, perla, coral, alga y arena.

Las transcripciones se entregaron a los participantes para su revisión y corrección, sin realizar modificaciones en el contenido. El método analítico consistió en visibilizar las relaciones que constituyen una realidad dada, en la cual la investigadora está inmersa. Las temáticas emergentes se identificaron a partir de la recopilación de datos, utilizando un guion de siete preguntas abiertas. 

## RESULTADOS

### La flexibilización en los procesos de trabajo: el *tratamiento directamente observado* (TDO) y la *visita domiciliaria* (VD) como prácticas de atención más allá del protocolo

Todas las preguntas del guion se relacionaban con el proceso de trabajo de los equipos de salud de las unidades entrevistadas. Cuando se les preguntó acerca de las estrategias que utilizaban los equipos de salud para garantizar el tratamiento de las personas usuarias con tuberculosis, las y los profesionales de la salud mencionaron las prácticas del *tratamiento directamente observado* (TDO) y la *visita domiciliaria* (VD), que son actividades protocolares, ya establecidas en la rutina de la atención primaria de salud, con formas y horarios flexibles.

*Utilizamos el tratamiento supervisado. Nuestra estrategia es brindar un buen acogimiento. Mostrarle al paciente la importancia de completar el tratamiento. También enfatizar que es muy importante que mantenga una rutina en la toma de la medicación. Él es quien elige un horario, selecciona un horario*... (Agente comunitario de Salud - Pez)

La flexibilización en la entrega de medicamentos -ya sea fuera del horario de funcionamiento de la unidad de salud, a un familiar o vecino, a la red familiar y comunitaria identificada en el territorio o para más de siete días- surge de los problemas reales experimentados por los equipos. Según la experiencia de los profesionales entrevistados, hubo casos de personas usuarias en tratamiento que no estaban en casa o tenían dificultades para ir a la unidad de salud para que se observara la toma diaria de medicamentos, como se recomienda. De esta manera, estas alternativas de atención para la entrega de medicamentos configuran la práctica de la atención primaria, que derivan de la necesidad de reorientar aquellas prácticas de atención que, aunque estén recomendadas en los protocolos, no lograban obtener un resultado favorable.

*...Ya es difícil hacerlo durante seis meses, y si no se hace de la manera adecuada, puede prolongar más este tratamiento. Intentamos mantener un vínculo con el paciente para que se sienta cómodo en el equipo. Facilitamos su acceso. Cuando no puede estar o ir a la unidad para recoger y tomar el medicamento, lo llevamos a su casa. Tratamos de acordar de la mejor manera posible lo que sería mejor para el paciente para que siga el tratamiento de manera adecuada*. (Técnico de enfermería - Agua)

*…Ofrecemos horarios y lugares flexibles. A veces, el usuario no se siente cómodo recibiendo visitas en casa, y proponemos ir a la unidad... En caso de que el usuario tenga limitaciones de horario debido al trabajo y no pueda ir a la unidad, es posible acordar otros lugares*. (Agente Comunitario - Caracol)

La estrategia de flexibilizar la rutina de visitas domiciliarias a usuarios sin dirección fija, o aquellos que trabajan de manera independiente y pasan la mayor parte del tiempo fuera de casa, o que están involucrados en el tráfico de drogas, son ejemplos de situaciones complejas que llevaron a los equipos a establecer alternativas para completar el proceso terapéutico de estos casos complejos, que a menudo interrumpen el tratamiento.

La presencia de términos como “acogida”, “acceso”, “vínculo” y “flexibilidad” en los discursos de los profesionales y en las historias presentes en los informes evidencian aspectos de la tecnología relacional entre el equipo de salud y el usuario, superando la lógica mecanicista de estas prácticas. Esto se debe a que estas acciones hablan sobre la gestión del proceso de trabajo desde la humanización de la asistencia, dado que la comunicación y la acogida son herramientas esenciales en el proceso de cuidar^25^.

A partir de preguntas desencadenantes, fue posible acceder a las experiencias compartidas por las personas entrevistadas que surgieron de la vida cotidiana, es decir, cargadas de la intensidad de los contenidos, eventos y afectos presentes en ese contexto. De esta manera, fue posible identificar “la experiencia en el discurso”^26^ a partir del uso de la cartografía. Esto se debe a que expresar los afectos tiende a modificar la realidad, ya que narrar es construir nuevas formas^27^. De esta manera, la cartografía, en el proceso de construcción colectiva de lo real, posibilita involucrar a los sujetos de la investigación en la reflexión sobre su realidad y luchar para dejar de ser objetos y convertirse también en sujetos portadores de testimonios y reflexiones sobre su propia condición poética, lingüística y política^28^.

Las acciones relatadas ponen de manifiesto que el trabajo vivo tiene como finalidad mejorar la comprensión de las acciones de atención establecidas, fortaleciendo así el vínculo de la persona usuaria con la unidad de atención, lo cual es esencial en el contexto del tratamiento de la tuberculosis, dada la extensa duración para lograr un resultado de alta por curación.

El enfoque del tratamiento directamente observado y la visita domiciliaria implica la formación y el fortalecimiento de vínculos, y aquí radica su importancia en la reducción de la interrupción, ya que implica la corresponsabilidad en el proceso de cuidado^29^. Al comprender que la educación permanente en salud surge de la experiencia práctica de las y los trabajadores en su vida cotidiana, se entiende que también se desarrolla en tecnologías ligeras, es decir, en las relaciones establecidas en estos encuentros de visitas domiciliarias y entrega de medicamentos, donde se generan conocimientos, saberes, intercambios y diálogos.

El uso de estas subjetividades está relacionado con la integralidad, equidad, acogida y vínculo, y todas las acciones deben ser utilizadas en la prestación de servicios, ya que permiten un conocimiento real de las necesidades específicas de cada individuo.

### Nuevas formas de cuidar a través del trabajo interdisciplinario

Cuando se les preguntó en qué actividades de cuidado realizadas en el contexto de las unidades de atención primaria fue posible identificar la práctica del “apoyo matricial” como una herramienta de educación permanente en salud desarrollada para el manejo de casos de interrupción del tratamiento, las personas entrevistadas mencionaron la práctica de compartir conocimientos, incluyendo la participación del personal de trabajo social en asuntos de beneficios sociales para las familias con el fin de obtener programas de transferencia de ingresos previstos por el gobierno federal, así como el apoyo psicológico, especialmente para los familiares que están sobrecargados en este proceso de enfermedad.

Por definición, la práctica del apoyo matricial se puede describir como una atención colaborativa que tiene un enfoque pedagógico-terapéutico y que se centra en la acción de los profesionales que forman parte del equipo de los núcleos de apoyo a la salud familiar (NASF). La creación de estos núcleos se estableció en 2008 con el propósito de proporcionar apoyo matricial en una variedad de cuestiones de salud, incluyendo la salud mental. En 2023, el Sistema Único de Salud (SUS) inició una reconstrucción y reformulación de esta estrategia de abordaje anteriormente denominada equipo NASF, que pasó a denominarse “eMulti”. Esta nueva propuesta retoma, innova y fortalece el cuidado multiprofesional en la atención primaria, basándose en la experiencia del NASF que era parte integral del SUS^30^.

El trabajo basado en la metodología del apoyo matricial amplía las posibilidades de producción del cuidado, permitiendo abordar la política de reducción de daños con las personas usuarias que han interrumpido el tratamiento y que consumen alcohol y otras drogas, la obtención de beneficios sociales cuando es necesario, y el seguimiento psicológico y nutricional, por ejemplo. En este proceso, discutir en los espacios de reuniones del equipo permite identificar los puntos débiles y las fortalezas en el caso de la interrupción y elaborar colectivamente, desde una perspectiva multiprofesional, las necesidades de apoyo en el proceso de atención para pensar nuevos caminos terapéuticos, considerando incluso la inclusión del usuario en este proceso: una práctica denominada *proyecto terapéutico singular*.

En la elaboración de un proyecto terapéutico singular es fundamental tener en cuenta los siguientes aspectos: los protocolos de atención y directrices clínicas que organizan la rutina de las consultas programadas y los exámenes necesarios; los significados y los valores que rodean cada caso, tanto para la persona usuaria como para el servicio de salud, así como el grado de responsabilidad de los miembros del equipo. Es importante que el plan terapéutico no sea elaborado únicamente por el equipo de salud, especialmente cuando se trata de tuberculosis, ya que evita que se convierta en una prescripción hecha solo con “orientación científica”, sin la participación de la persona que padece la enfermedad, que no se siente considerado en sus deseos y expectativas^31^.

En las entrevistas realizadas se identificó una división de tareas en el proceso de trabajo de los equipos de salud y la reunión del equipo que se lleva a cabo semanalmente, momento que sirve para reevaluar la planificación y revisar metas y plazos. En la dinámica del proceso de atención, el agente comunitario de salud suele ser la persona responsable de la observación de la toma de medicamentos, así como de las visitas domiciliarias; el farmacéutico actúa como la persona encargada de dispensar medicamentos y verifica el cambio de fase del tratamiento en el período recomendado; el profesional de enfermería se encarga del seguimiento de la evaluación clínica, y también se tiene en cuenta la preferencia de la persona usuaria en cuanto al horario de las citas y la forma de entrega de la medicación.

Aunque este proceso de división de etapas, reevaluación y planificación no está presente en todos los casos de interrupción del tratamiento, es posible identificar que las y los profesionales de los equipos de salud están implementando el proyecto terapéutico singular como una herramienta de trabajo, al considerar las opiniones de la persona usuaria para determinar la mejor manera de llevar a cabo el seguimiento, el horario o el lugar para las consultas, y al utilizar las reuniones del equipo para identificar quién tiene un mayor vínculo para reanudar el seguimiento en casos de interrupción.

A lo largo de las entrevistas, ninguno de los profesionales utilizó esta nomenclatura para denominar las acciones realizadas con relación al proyecto terapéutico singular. Este hallazgo destaca la necesidad de fomentar espacios de discusión de casos complejos a partir de la teoría fundamentada en la práctica y las herramientas de atención primaria, como el familiograma, el genograma y el ecomapa, con el fin de capacitar aún más a los profesionales para brindar atención centrada en la persona.

### La mejoría clínica y el uso de alcohol y otras drogas como predictores de la interrupción del tratamiento de la tuberculosis

Sobre las dificultades de los equipos de salud en el abordaje a las personas usuarias que interrumpieron el tratamiento de la tuberculosis, los entrevistados señalaron cuestiones relacionadas con la percepción de la mejora clínica y la interrupción voluntaria por parte del usuario, cambios en el territorio que dificultan la supervisión por parte del equipo, y el consumo de alcohol y drogas, es decir, aspectos que involucran situaciones del usuario.

…*Las dificultades con respecto al enfoque, creo que se centran en convencer al paciente de que debe completar los seis meses de tratamiento, incluso si ya se siente saludable, para que continúe tomando la medicación, que acuda a la unidad o que nosotros la llevemos hasta su residencia, ya que a menudo argumentan esto cuando abandonan el tratamiento: “ya estoy bien, así que no necesito más tratamiento”*. (Enfermero - Perla)

*La dificultad en el enfoque es convencer al paciente de que regrese. Después de que el paciente ya no tiene síntomas, quiere detener el tratamiento, piensa que ya está curado, dice que ya ha pasado mucho tiempo. Entonces, las dificultades que enfrentamos es que pasan dos o tres meses y piensan que ya están bien. Y tratamos de mostrarles hasta el final que el tratamiento es a largo plazo y debe ser completado durante los seis meses*. (Farmacéutico - Ostra)

*El paciente, al percibir una mejoría en el cuadro clínico, cree que está curado y se resiste a la orientación de seguir con el tratamiento.* (Técnico de enfermería - Arena)

*Tenemos casos en los que interrumpen el tratamiento varias veces y, cuando se sienten mal, vuelven. Llevé a la enfermera varias veces a hablar con él, le llevaba los medicamentos en la mano, trataba de verlo mientras los tomaba, pero desafortunadamente no completó el tratamiento, hasta hoy. Llevé a la enfermera nuevamente a su casa hasta que se mudó. Ahora se mudó a otra área y no está recibiendo tratamiento*. (Agente Comunitario - Alga)

Otras de las dificultades señaladas por los equipos de salud con relación a la interrupción del tratamiento se refieren tanto a cambios en el territorio, que dificultan la supervisión por parte del equipo, como al consumo de alcohol y drogas, es decir, aspectos que involucran situaciones de las personas usuarias.

*La dificultad en el abordaje ocurrió con un caso en mi área. Él mentía mucho. Decía que tomaba, pero su esposa decía que era mentira. Iba allí varias veces y lo veía vomitar sangre, con fiebre porque interrumpía el tratamiento... Hablamos, pero no podemos hacer mucho más, ¿verdad? Quiero decir, hacíamos mucho llevándole la medicación en la mano. ¡Ah! ¡Y tenía muchos medicamentos! Es decir, dejaba todos los medicamentos en su casa y no los tomaba. Además de la bebida, además de la droga, ya que es usuario de drogas. El abordaje es realmente complicado*. (Agente Comunitaria de Salud - Coral)

*Los usuarios de drogas y los involucrados en el tráfico son difíciles de tratar, porque son itinerantes. El equipo tiene dificultades para encontrarlos en el territorio*. (Enfermero - Onda)

*Después de buscar al paciente en la unidad y obtener resultados positivos en sus exámenes, se asigna al agente comunitario de salud responsable de esa dirección y se realiza una visita domiciliaria para la búsqueda activa del paciente. En muchas ocasiones, no se logra localizar al paciente debido a la dificultad para encontrar la dirección. Por ejemplo: calles sin nombres, viviendas sin numeración, a menudo con números de teléfono fuera de área, vecinos que solo conocen al paciente por apodos o nombre social y no por su nombre real.* (Enfermero - Playa)

Las normas de la Estrategia de Salud Familiar (ESF) tienen como atributo estandarizar las prácticas de las y los trabajadores de acuerdo con las reglas establecidas para el funcionamiento del programa. Sin embargo, estas normas influyen en la actividad de los trabajadores dentro de límites muy estrechos. Cuando se encuentran en situación de trabajo, en relación con el usuario, son ellos mismos, en el acto de trabajar, quienes definen cómo se llevará a cabo el cuidado^14^.

Hoy en día, el Sistema Único de Salud (SUS) sigue enfrentando desafíos para su plena implementación, que incluyen: la fragmentación del proceso de trabajo entre diferentes profesionales; un sistema público centralizado y burocratizado; la falta de preparación para abordar la dimensión subjetiva en las prácticas de atención; la precarización de la gestión participativa y la debilidad del control social; la formación profesional alejada de la actuación política; y un modelo de atención centrado en la relación entre queja y conducta^32^.

Estas lagunas se hacen evidentes, sobre todo en la revisión de la bibliografía, que señala críticas respecto de la incapacidad de esta estrategia para prevenir el aumento de la incidencia de la tuberculosis en áreas con una alta prevalencia. Estudios realizados en los municipios de Porto Alegre, Río de Janeiro y São Paulo muestran que la prevalencia de la tuberculosis es alta en la población sin hogar y puede ser hasta 70 veces mayor que en la población general^33^. En esta población, también son más comunes los resultados desfavorables, como un bajo porcentaje de curación, una alta tasa de interrupción del tratamiento (alrededor de cuatro veces mayor que en la población general) y una proporción elevada de muertes entre los casos notificados^8^.

El boletín epidemiológico publicado en marzo de 2023, que analiza la tuberculosis en el municipio de Río de Janeiro, señala que la distribución espacial de los casos nuevos en 2022 tiene una correlación con la distribución socioeconómica de la población carioca. Se pueden observar altas concentraciones en comunidades densamente pobladas y de gran contingente poblacional, entre ellas el complejo de Maré, escenario de investigación de este artículo^34^.

Los desafíos asociados con la gestión de los casos de tuberculosis demuestran que la atención va más allá del ámbito biomédico. El cumplimiento de las directrices técnicas es insuficiente para lograr el éxito en el control de la enfermedad, ya que se deben tener en cuenta los aspectos sociales, económicos y el contexto de los servicios de salud donde se llevarán a cabo las acciones.

## DISCUSIÓN

Los relatos recopilados surgen de la experiencia vivida por las y los profesionales de las unidades de salud de Maré. A lo largo del análisis, fue posible identificar el potencial y las limitaciones en el proceso de atención de los usuarios con tuberculosis, así como la construcción de relaciones y sus posibilidades de apoyo en el manejo de casos complejos y de aquellos que interrumpieron el tratamiento de la tuberculosis.

La comprensión del proceso que implica la interrupción del tratamiento de la tuberculosis abarca la discusión sobre la adhesión, que va más allá del simple acto de tomar la medicación, ya que está fuertemente relacionada con la construcción de subjetividades, es decir, cómo el paciente comprende la enfermedad y cómo se relaciona con los servicios de salud^31^.

Al flexibilizar la forma en que se puede llevar a cabo la práctica del tratamiento directamente observado y las visitas domiciliarias, al buscar apoyo en la red comunitaria, al fomentar la discusión en equipo y considerar la opinión del usuario en su proceso de atención, se rompe con la verticalidad del conocimiento y se abre espacio para la creatividad, superando las respuestas normativas. De esta manera, es posible comprender el tratamiento directamente observado y las visitas domiciliarias en esta investigación no solo como herramientas exclusivas para curar la enfermedad, sino también como una forma de garantizar la atención y la medicación de manera más adecuada a las necesidades y complejidades de cada paciente.

La micropolítica de la gestión y la atención médica en atención primaria de la salud indica que todas las partes llegan informadas y con una idea sobre lo que debería suceder en ese encuentro: la producción de un diagnóstico, escuchar una aflicción, una intervención que resuelva el problema o alivie el sufrimiento, la liberación del usuario y otros aspectos. Además, señala que el encuentro está lleno de expectativas y mutuas interferencias que otorgan un carácter de imprevisibilidad al resultado del trabajo en salud. Dado que el encuentro se produce en acto, es parcialmente incontrolable^12^.

Hemos identificado que todo este proceso de construcción colectiva, en busca de alternativas para resolver casos complejos, a veces pasa desapercibido en las actividades de los equipos. No es reconocido ni por los propios profesionales, que no se ven en un proceso continuo de educación permanente. Es importante destacar que la práctica de la educación permanente en salud ocurre en el día a día, entre los trabajadores y los usuarios, donde se generan nuevos procesos colectivos que no se habían buscado hasta ese momento. En otras palabras, reinventar el trabajo con ajustes en la forma de abordar los problemas consiste en el potencial transformador de la educación permanente en salud y no necesita necesariamente un espacio formal para la transferencia de conocimientos.

Por lo tanto, es fundamental considerar que el aprendizaje en el trabajo, a través del trabajo y para el trabajo, se construye a partir de los problemas que se enfrentan en la realidad de los servicios. La educación permanente en salud va más allá de la acción educativa con momentos formales en un aula con información técnicamente orientada. Ocurre en los encuentros, en los espacios de discusión colectiva, con el objetivo de ampliar la capacidad de acción de todos los involucrados en el equipo, incluido, y especialmente, la propia persona usuaria en cuestión.

Respecto al fenómeno de la adhesión al tratamiento y la atención en los servicios de salud, a menudo los equipos de salud multiprofesionales no comprenden que las personas usuarias no sigan el tratamiento y se sienten impotentes^32^.

Por lo tanto, la educación permanente en salud se vuelve fundamental para llevar a los profesionales que trabajan en atención primaria de la salud a reflexionar sobre sus problemas y a identificar nuevas formas de brindar atención de acuerdo con las limitaciones y capacidades de la población atendida. Es esencial que el servicio de salud supere la lógica de culpar a la persona usuaria por interrumpir el tratamiento y se posicione como un proceso de coparticipación en el cual el servicio de salud también debe estar disponible y ser un colaborador en este proceso de atención.

## CONSIDERACIONES FINALES

Al analizar los diferentes acuerdos locales para la producción de educación permanente en salud, esta investigación buscó dar espacio al trabajo creativo de los equipos de salud, así como a sus fortalezas y desafíos en el manejo de casos complejos de tuberculosis. En este sentido, se pudieron identificar herramientas y prácticas cotidianas que se están implementando en las diferentes unidades de salud de la comunidad de Maré en Río de Janeiro.

El campo de las tecnologías de atención se reveló como un lugar privilegiado para arrojar luz sobre las prácticas de educación permanente en los servicios de salud. A través de este enfoque, se les dio voz a los profesionales para que presentaran diversas formas de involucrarse en el acto de cuidar, sus enfoques para abordar las situaciones que enfrentan, así como sus dificultades y fortalezas. Este proceso se basó en la memoria y la problematización de los casos complejos que habían experimentado.

Esta materialización de las experiencias vividas y compartidas se facilitó a través del proceso de la cartografía en salud, ya que permitió el mapeo del panorama de los procesos de atención al usuario en interrupción de tratamiento, al mismo tiempo que analizaba la educación permanente en las unidades de salud estudiadas.

Los profesionales de atención primaria están inmersos en la complejidad cotidiana de la atención a la población residente en la comunidad de Maré. A pesar de esto, se produce un continuo proceso de resiliencia, problematización de la realidad vivida y construcción de conocimientos para planificar cuidados alternativos cuando es necesario.

Los resultados de la investigación identificaron que se llevan a cabo prácticas de educación permanente en todos los espacios de las unidades de salud visitadas. Es importante destacar que la educación permanente en salud no se establece como un mero espacio informativo o un entrenamiento destinado a aumentar las habilidades específicas de sus participantes, sino como un proceso en el que se afirma la relación inseparable entre el aprendizaje en salud y el trabajo en salud.

Sin embargo, se puede observar que las y los profesionales no reconocen el potencial que poseen las acciones que realizan, ni que los actos productivos constituyen educación permanente en salud. A menudo creen que es necesario estar en un espacio formal con intercambio de conocimientos protocolarios, sin valorar la rica construcción que se lleva a cabo en los pasillos de la unidad de salud, en las reuniones de equipo, en las calles del territorio, en las casas de los usuarios, que permite la creatividad y la flexibilidad de los procesos de atención más allá de las prácticas normativas.

También se identificaron dificultades enfrentadas por los servicios de salud de atención primaria, como la limitación para implementar y poner en práctica las herramientas de la educación permanente en salud. La precariedad de los vínculos laborales y la dificultad para asignar médicos en áreas violentas de Maré, que afectan negativamente la composición de los equipos de salud, así como la composición del equipo multiprofesional (eMulti), dificultan el proceso de construcción colectiva.

Además, los equipos de salud a menudo ven que sus planes se ven perjudicados por otras demandas del servicio, especialmente en los últimos dos años durante la pandemia de covid-19. Estas circunstancias a veces limitan el proceso de comunicación entre las y los profesionales en los servicios debido a la carga de trabajo intensa, así como a la práctica de vigilancia del equipo. Esto resulta en la falta de tiempo para discutir y reflexionar sobre proyectos terapéuticos, lo que tiene un impacto en los indicadores de subnotificación de nuevos casos y en los casos que interrumpen el tratamiento, que el equipo no puede identificar y abordar para su reincorporación.

Como potencialidad, la investigación permite que las y los trabajadores profundicen su conocimiento sobre la teoría de las herramientas que ya utilizan en su vida cotidiana, yendo más allá del proceso reflexivo sobre el mundo laboral de los sujetos. Reconocer las expresiones de la educación permanente en salud en y a través del trabajo requiere esfuerzo, salir de la zona de confort y ampliar las posibilidades de respuestas dirigidas a una mayor resolutividad y calidad de las necesidades de salud de los servicios y las personas usuarias.

La mejora del indicador de abandono del tratamiento de la tuberculosis sigue siendo un problema de salud pública y suscita la reflexión sobre la impotencia de las acciones protocolares que ya están en marcha y que, aun así, no son suficientes para resolverlo. Esto se debe a que las políticas no priorizan este trabajo vivo de interrogación y reflexión. Por lo tanto, se recomienda que se realicen más investigaciones para dar voz a las y los profesionales de la salud, para destacar nuevos enfoques que se están construyendo en el trabajo cotidiano en las unidades de salud, ya que es a partir de la problematización de la realidad vivida en las consultas de salud, en el territorio o en la ausencia del usuario en las citas, que los equipos reflexionan y organizan nuevas estrategias de atención al usuario.

Se espera que, a partir de las reflexiones promovidas a lo largo de las entrevistas de esta investigación, los trabajadores en acción sientan estímulo para continuar utilizando las herramientas que ya utilizan y reconozcan, a través de la construcción de vínculos, la importancia de la atención y el cuidado ofrecidos y la potencia de las reinversiones y flexibilidades en su vida cotidiana frente a la complejidad de la realidad que viven y comparten con los usuarios en sus territorios. Así, esta investigación se convierte en un medio para arrojar luz sobre lo que se hace y quiénes realizan el trabajo.

Finalmente, identificamos la necesidad de que las y los profesionales de la salud reconozcan el potencial que representa la educación permanente en salud, para que sea vivida por los trabajadores en acción y valorada. A pesar de ser invisibilizada, la educación permanente en salud ocurre día tras día, con creatividad y flexibilidad, en la dinámica de las relaciones, ya sea en los pasillos de las unidades de salud o en el territorio de las comunidades, se produce en el trabajo vivo en acto.
